# Genomic insights into the cold adaptation and secondary metabolite potential of *Pseudoalteromonas* sp. WY3 from Antarctic krill

**DOI:** 10.3389/fmicb.2024.1459716

**Published:** 2024-11-05

**Authors:** Yuanyuan Wang, Jinxuan Xie, Zhengqi Feng, Linbo Ma, Wenbo Wu, Changjun Guo, Jianguo He

**Affiliations:** 1 State Key Laboratory for Biocontrol/Southern Marine Science and Engineering Guangdong Laboratory (Zhuhai), Guangdong Province Key Laboratory of Aquatic Economic Animals & Guangdong Provincial Observation and Research Station for Marine Ranching of the Lingdingyang Bay, School of Marine Sciences, Sun Yat-sen University, Guangzhou, China; 2 Key Laboratory of the East China Sea and Oceanic Fishery Resources Exploitation, Ministry of Agriculture, East China Sea Fisheries Research Institute, Shanghai, China

**Keywords:** Antarctic krill, marine bacterium, *Pseudoalteromonas*, genomic analysis, extremophiles

## Abstract

In the Antarctic marine ecosystem, krill play a pivotal role, yet the intricate microbial community intertwined with these diminutive crustaceans remains largely unmapped. In this study, we successfully isolated and characterized a unique bacterial strain, *Pseudoalteromonas* sp. WY3, from Antarctic krill. Genomic analysis revealed that WY3 harbors a multitude of genes associated with cold shock proteins, oxidoreductases, and enzymes involved in the osmotic stress response, equipping it with a robust molecular arsenal to withstand frigid Antarctic conditions. Furthermore, the presence of two distinct biosynthesis-related gene clusters suggests that WY3 has the potential to synthesize diverse secondary metabolites, including aryl polyenes and ribosomally synthesized and post-translationally modified peptides. Notably, the identification of genes encoding enzymes crucial for biological immunity pathways, such as *apeH* and *ubiC*, hints at a complex symbiotic relationship between WY3 and its krill host. This comprehensive study highlights the robust potential of WY3 for secondary metabolite production and its remarkable ability to thrive at extremely low temperatures in the Antarctic ecosystem, shedding light on the interplay between culturable microorganisms and their hosts in harsh environments, and providing insights into the underexplored microbial communities associated with Antarctic marine organisms and their role in environmental adaptation and biotechnological applications.

## 1 Introduction

The Antarctic krill (*Euphausia superba*) plays a pivotal role in the Southern Ocean ecosystem, serving as a crucial link between primary producers and higher trophic levels (Croxall et al., 1999). While extensive research has been conducted on the bioactive compounds of krill (Tou et al., 2007) and their contributions to biogeochemical cycling (Cavan et al., 2019), the complex microbial communities associated with these crustaceans remain largely unexplored. Understanding these host–microbe interactions is essential for comprehending the ecological dynamics and potential biotechnological applications of krill-associated microorganisms.

Among the diverse microorganisms inhabiting marine environments, the bacterial genus *Pseudoalteromonas* has emerged as a subject of significant interest since its description in 1995 (Gauthier et al., 1995). These strictly aerobic, heterotrophic, gram-negative bacteria belonging to the marine gamma-proteobacteria family are characterized by genomic G + C contents of 38–48 mol% (Hwang et al., 2016). *Pseudoalteromona*s species are frequently isolated from various extreme marine habitats, including the Arctic (Liao et al., 2019), Antarctic (Colarusso et al., 2022) and deep-sea sediments (Qin et al., 2011). In addition to their presence in extreme environments, *Pseudoalteromonas* species are frequently associated with higher marine organisms, including algae (Xu et al., 2021), invertebrates (Cuny et al., 2021) and fish (Kawase et al., 2022). Such associations are particularly noteworthy in polar environments, where symbiotic relationships often contribute to survival under extreme conditions.

The adaptation of *Pseudoalteromonas* to cold environments, particularly in polar regions, is a subject of growing research interest. Polar bacteria have evolved various cold adaptation mechanisms to thrive at subzero temperatures, a defining feature of polar habitats. Key adaptations include the production of cold-active enzymes, antifreeze proteins, and cryoprotectants that enable cellular functions and metabolic activities to continue in freezing conditions (De Maayer et al., 2014). Cold-active enzymes are structurally adapted to function optimally at low temperatures by maintaining flexibility at active sites, which is essential for catalytic efficiency (Cavicchioli et al., 2002). Additionally, the production of antifreeze proteins helps prevent the formation of ice crystals that can damage cellular structures (DeVries and Cheng, 2005).

In addition to these physiological adaptations, membrane flexibility plays a crucial role in the survival of polar bacteria. The ability to synthesize polyunsaturated fatty acids (PUFAs) and alter membrane lipid composition ensures membrane fluidity at freezing temperatures (Chattopadhyay, 2006). This flexibility is vital for nutrient uptake and waste expulsion, processes that are compromised at low temperatures in organisms with rigid membranes. Furthermore, polar bacteria, including *Pseudoalteromonas*, often produce extracellular polysaccharides and other secondary metabolites that play a role in the stress response and protection against environmental challenges, such as freezing, oxidative stress, and competition with other microorganisms (Holmström and Kjelleberg, 1999).

Symbiotic relationships between *Pseudoalteromonas* species and marine organisms have been documented, particularly in the context of providing defense mechanisms to their hosts. For example, *Pseudoalteromonas* species associated with marine algae produce bioactive compounds that inhibit fouling organisms, thereby benefiting algae by reducing competition for light and nutrients (Xu et al., 2021). Similar protective roles have been observed in symbioses with invertebrates and fish (Cuny et al., 2021; Kawase et al., 2022). These bioactive compounds, often secondary metabolites, include antibiotics, antimicrobials, and antifouling agents that are crucial for mediating host–microbe interactions in harsh marine environments. The ability to produce such metabolites enhances the survival of both bacteria and their hosts in extreme environments.

Despite these findings, the specific interactions between *Pseudoalteromonas* and their Antarctic krill hosts remain largely unexplored. This represents a significant gap in our understanding of marine microbiomes in polar ecosystems. The presence of biosynthesis-related gene clusters (BGCs) in *Pseudoalteromonas* species, which are responsible for synthesizing diverse secondary metabolites, suggests that these bacteria may contribute to the defense and survival of their krill hosts in the extreme Antarctic environment (Bowman, 2007; Chau et al., 2021). These BGCs may play a role in providing protective compounds or assisting in nutrient acquisition, thereby facilitating a mutually beneficial relationship between bacteria and krill.

In the present study, *Pseudoalteromonas* sp. strain WY3 was isolated from Antarctic krill, with the aim of revealing its ecological significance and biotechnological potential through comprehensive taxonomic, phylogenetic, chemotaxonomic, whole-genome, and comparative genomic analyses. This study provides valuable insights into the symbiotic relationships between *Pseudoalteromonas* and Antarctic krill and highlights the importance of synthetic gene clusters in cold adaptation and microbial–host interactions in extreme environments. This research contributes to the broader understanding of polar biology and the potential for novel biotechnological applications derived from cold-adapted microorganisms by expanding our knowledge of these microbial communities.

## 2 Materials and methods

### 2.1 Isolation of *Pseudoalteromonas* sp. WY3

Antarctic krill samples (58–60° W, 62–64° S) were collected from the East China Sea Fisheries Research Institute (Shanghai, China) in 2016 (Wang et al., 2022). Sample processing was carried out according to our previous methods (Feng et al., 2023; Wang et al., 2021). The isolation of *Pseudoalteromonas* sp. strain WY3 was carried out under sterile conditions to ensure the purity of the bacterial culture. Initially, the krill samples were rinsed with sterile seawater to remove loosely attached microorganisms. The samples were subsequently homogenized in sterile seawater to create a suspension. Aliquots of the suspension were then serially diluted and spread onto Luria–Bertani (LB), a medium specifically formulated for the cultivation of marine bacteria, containing the following components per liter: 5 g of peptone, 1 g of yeast extract, 15 g of agar, and 500 mL of natural seawater, with the final volume adjusted to 1 L using distilled water. The pH of the medium was adjusted to 7.6. The plates were incubated at 10°C for 5–7 days to allow for the growth of cold-adapted microorganisms. Distinct colonies were picked based on morphological characteristics and streaked onto fresh marine agar plates to obtain pure cultures. For long-term storage, sterile glycerol was added to the liquid medium (25%, v/v), which was subsequently stored in a refrigerator at −80°C.

### 2.2 Growth curve determination

To characterize *Pseudoalteromonas* sp. strain WY3 comprehensively, the isolate was cultured in LB media across a range of temperatures (4°C, 8°C, 12°C, 16°C, and 25°C) to assess its growth dynamics under different environmental conditions. The cultures were incubated with continuous agitation at 150 rpm to ensure optimal aeration. Growth was monitored by measuring the optical density at 600 nm (OD600) at regular intervals via a spectrophotometer. Bacterial growth curves were generated by plotting the logarithm of OD600 readings on the y-axis against incubation time on the x-axis. To predict the psychrotolerant nature of WY3 and estimate its minimum and optimal growth temperatures, we employed the Ratkowsky model (Ratkowsky et al., 1983). The model equation is expressed as:


$$\surd r=b\;\left(T-T_{\min}\right)\;\left[1–\exp\;\left(c\;\left(T-T_{\max}\right)\right)\right]$$

where *T_min_* and *T_max_* represent the minimum and maximum temperatures, respectively, at which the growth rate is zero; *r* denotes the growth rate; *T* is the temperature; and *b* and c are regression coefficients.

### 2.3 Transmission electron microscope (TEM) observation

The morphological characteristics of *Pseudoalteromonas* sp. strain WY3 were examined via TEM. For sample preparation, a small amount of bacterial biomass was carefully collected from an overnight culture via a sterile inoculation loop and transferred to a 1.5 mL microcentrifuge tube. The bacterial suspension was washed three times with sterile ultrapure water, each time by centrifugation at 5000 × g for 5 min to remove any residual media components. Following the final wash, 10 μL of the bacterial suspension was carefully deposited onto a carbon-coated copper grid and allowed to adsorb for 1–2 min. Excess liquid was blotted off gently with filter paper. The sample was then negatively stained with 2% (w/v) phosphotungstic acid solution (pH 7.0) for 1 min to enhance contrast for electron microscopy. After air-drying, the grid was observed under a transmission electron microscope. The TEM images were used to analyze the bacterial cell shape, size, and surface structures.

### 2.4 DNA extraction and PCR amplification

Genomic DNA was extracted from the purified and cultured strains via a bacterial DNA extraction kit (Omega, Washington, DC, USA). The extracted DNA of the strain was used as a template to amplify the 16S rDNA region in bacteria with the universal primers 27F (5’-AGAGTTTGATCCTGGCTCA-3′) and 1492R (5’-GGTTACCTTGTTACGACTT-3′) (Tsingke, Beijing, China). The 16S rRNA gene sequence of strain WY3 was amplified and sequenced through the sequencing DNA service of TSINGKE Biological Technology, China.

### 2.5 Phylogenetic tree construction and genome assembly

The 16S rRNA gene sequence of strain WY3 was compared with the EzBioCloud database (Yoon et al., 2017), and ClustalW (Thompson et al., 1997) was used to analyze the phylogeny. The neighbor–joining phylogenetic tree was established via MEGA-X (Kumar et al., 2018), and the robustness of the phylogenetic tree was evaluated via bootstrap analysis (1,000 replicates) (Felsenstein, 1985). Single-molecule real-time (SMRT) sequencing was performed on the PacBio Sequel platform (Chin et al., 2013). PacBio sequencing and analysis were carried out by OE Biotech Co., Ltd. (Shanghai, China). Based on three generations of sequencing data, the genome was assembled *de novo*. Falcon was used for initial assembly, and the sequences were then corrected by GenomicConsensus software v0.8.0 (Chin et al., 2016). The SPRAI (single-pass read accuracy improver)-corrected sequences were used as auxiliary data for Circulator (Hunt et al., 2015) cyclization processing. Finally, a cyclized bacterial genome was obtained. The genome information of the bacteria has been uploaded to NCBI, and the accession number is JAAMPS000000000 for *Pseudoalteromonas* sp. WY3.

### 2.6 Genomic bioinformatics analysis

Coding gene prediction was performed via Prodigal (Prokaryotic Dynamic Programming Genefinding Algorithm) (v2.6.3) software (Hyatt et al., 2010). The repeat sequence was predicted via RepeatMasker v4.0.7 (Chen, 2004). tRNAscan-SE v1.3.1 (Lowe and Eddy, 1997) was used to predict tRNAs, RNAmmer v1.2 (Lagesen et al., 2007) was used to predict rRNAs, and Rfam v10.0 (Griffiths-Jones et al., 2003) was used to predict sRNAs. The default parameters were used for the preceding software. A draft genome map was generated via Circos (v0.69) software (Krzywinski et al., 2009). The digital DNA–DNA hybridization (dDDH) values were calculated via genome–BLAST Distance Phylogeny (GBDP) (Meier-Kolthoff et al., 2013). The average nucleotide identity (ANIb) value between genomes was calculated via the JSpeciesWS online service (Richter et al., 2016). Annotation was carried out via the Rapid Annotation using Subsystem Technology (RAST) server 2. 0 (Overbeek et al., 2014). Common database annotations were then performed. For NR (nonredundant protein), COG (Clusters of Orthologous Groups), GO (Gene Ontology), Swiss–Prot, eggNOG (Evolutionary genealogy of genes: Nonsupervised Orthologous Groups), and KEGG (Kyoto Encyclopedia of Genes and Genomes) database annotations, Diamond (Buchfink et al., 2015) software was used to compare the annotations with e < 1e-5, and the proteins with the highest sequence similarity were screened to obtain functional annotation information. The Pfam database is a collection of a series of protein families, and HMMER3 (Mistry et al., 2013) software was used for comparison with the protein family models in the Pfam database. HMMER (Mistry et al., 2013) software was used for comparison with the CAZy (Carbohydrate–Active enZYmes) (Cantarel et al., 2009) database. Genomic islands (GIs) were predicted via IslandViewer 4 (Bertelli et al., 2017). Biosynthesis-related gene cluster and secondary metabolic analyses were performed via the AntiSMASH server (Blin et al., 2019).

To compare the genomes, some reference genome sequences were downloaded from the GenBank database. Genomic homology cluster analysis was performed via OrthoVenn3 server (Sun et al., 2023). Venn diagrams were used to characterize the comparative genomic analysis of the genome of this bacterium and its related species.

## 3 Results

### 3.1 Isolation, identification, and phylogenetic analysis

**Figure 1:**
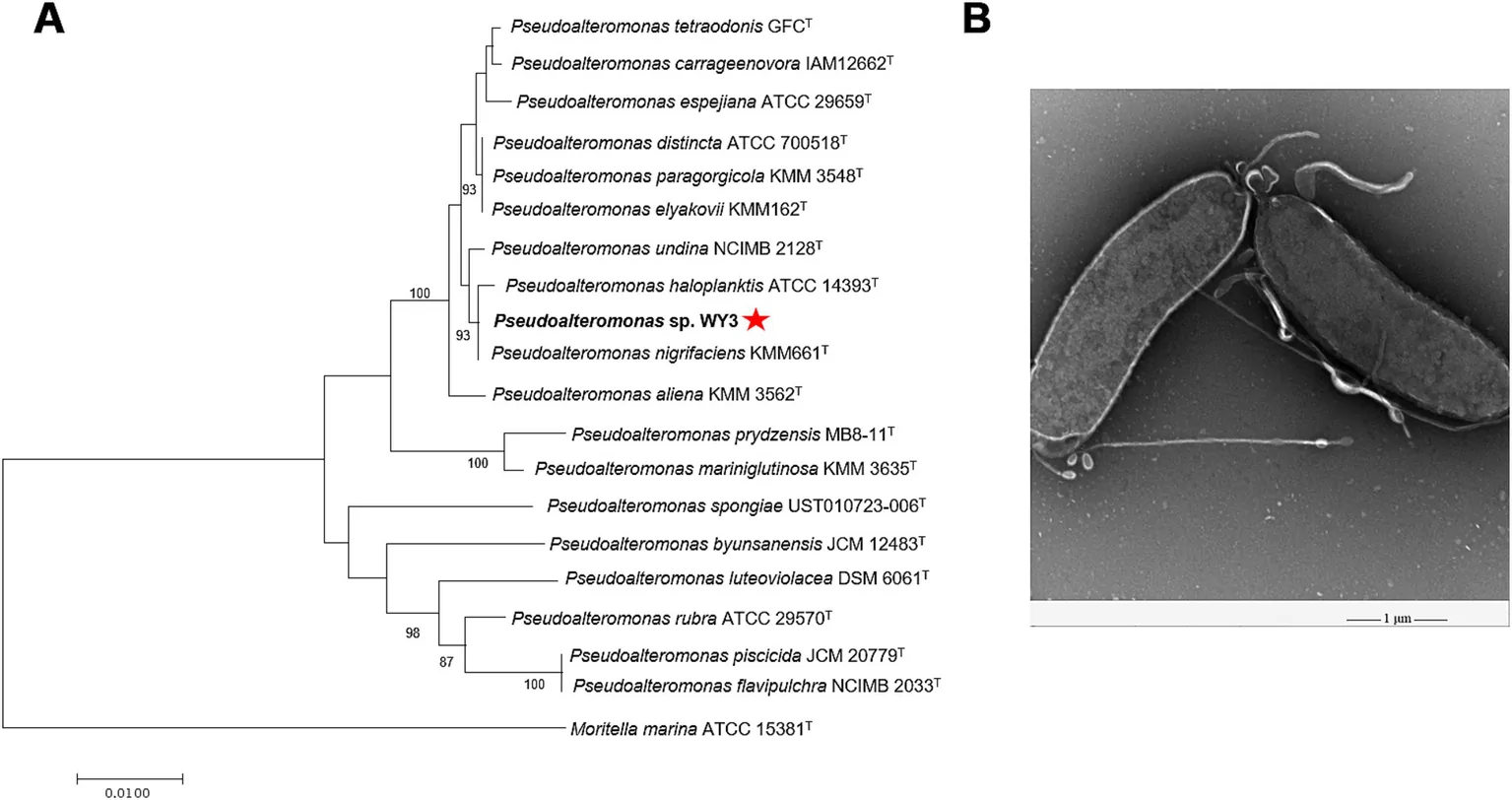
**(A)** The N-J tree shows *Pseudoalteromonas* sp. WY3 at the position of the concerned taxa on the 16S rRNA gene. Bar, 0.01 substitutions per nucleotide position. **(B)** Transmission electron micrograph showing cells of strain *Pseudoalteromonas* sp. WY3. The magnification is 5,000×. Bar, 1 μm.

The bacterial strain WY3 was successfully isolated from Antarctic krill via LB agar. Taxonomic classification based on the 16S rRNA sequence analysis revealed that strain WY3 belongs to the genus *Pseudoalteromonas*, with sequence similarity to *P. nigrifaciens* KMM661^T^, *P. haloplanktis* ATCC® 14393^T^ and *P. undina* NCIMB 2128^T^ (99.93, 99.86, and 99.79%, respectively) (Supplementary Table S1). Furthermore, 16S rRNA phylogenetic analysis confirmed the clustering of strain WY3 with certain nonpigmented species of the genus *Pseudoalteromonas* (Figure 1A). EM revealed that strain WY3 cells are rod shaped, with parallel sides and rounded ends, measuring approximately 2–3 μm in length and 0.70–0.95 μm in diameter (Figure 1B). These results establish strain WY3 as a member of the genus *Pseudoalteromonas*.

### 3.2 Genomic characteristics

**Figure 2:**
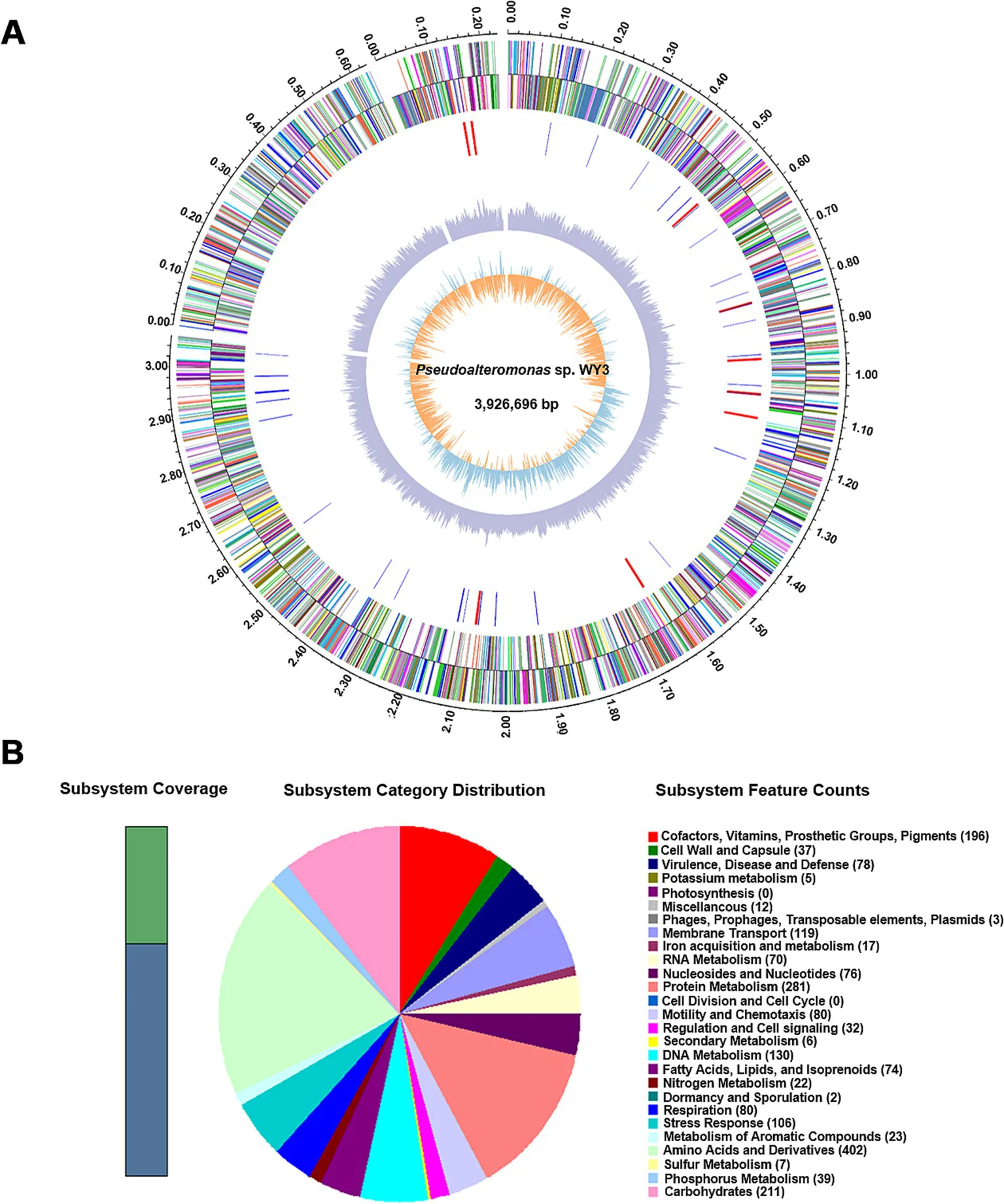
A draft map of the whole genome and the distribution of annotated genes of the *Pseudoalteromonas* sp. *WY3 strain*. **(A)** Genomic circle diagram of strain WY3. **(B)** *Pseudoalteromonas* sp. genome information annotated via the RAST server.

The genome assembly of strain WY3 included 3 scaffolds, with a total length of 3,926,696 base pairs and a G + C content of 39.99% (Table 1). A total of 4,234 genes were identified, including 4,107 coding sequences (Figure 2A). Functional annotation via the SEED viewer via the RAST pipeline categorized these genes into 27 subsystems (Figure 2B and Supplementary Table S2), with prominent subsystems related to amino acids and derivatives (402 genes), protein metabolism (281 genes), and carbohydrate metabolism (211 genes).

**Table 1:** Genome statistics of the *Pseudoalteromonas* sp. WY3.

Attribute	Value	% of total
Genome size (bp)	3,926,696	100
DNA coding region (bp)	3,315,162	84.43
DNA G + C (bp)	1,570,447	39.99
DNA scaffolds	3	–
Total genes	4,234	100
Protein coding genes	4,107	97.00
RNA gene	127	3.00
Genes assigned to COGs	3,059	74.48
Genes assigned Pfam domains	14	0.34
Genes with signal peptides	368	8.96
Genes with transmembrane helices	943	22.96

**Figure 3:**
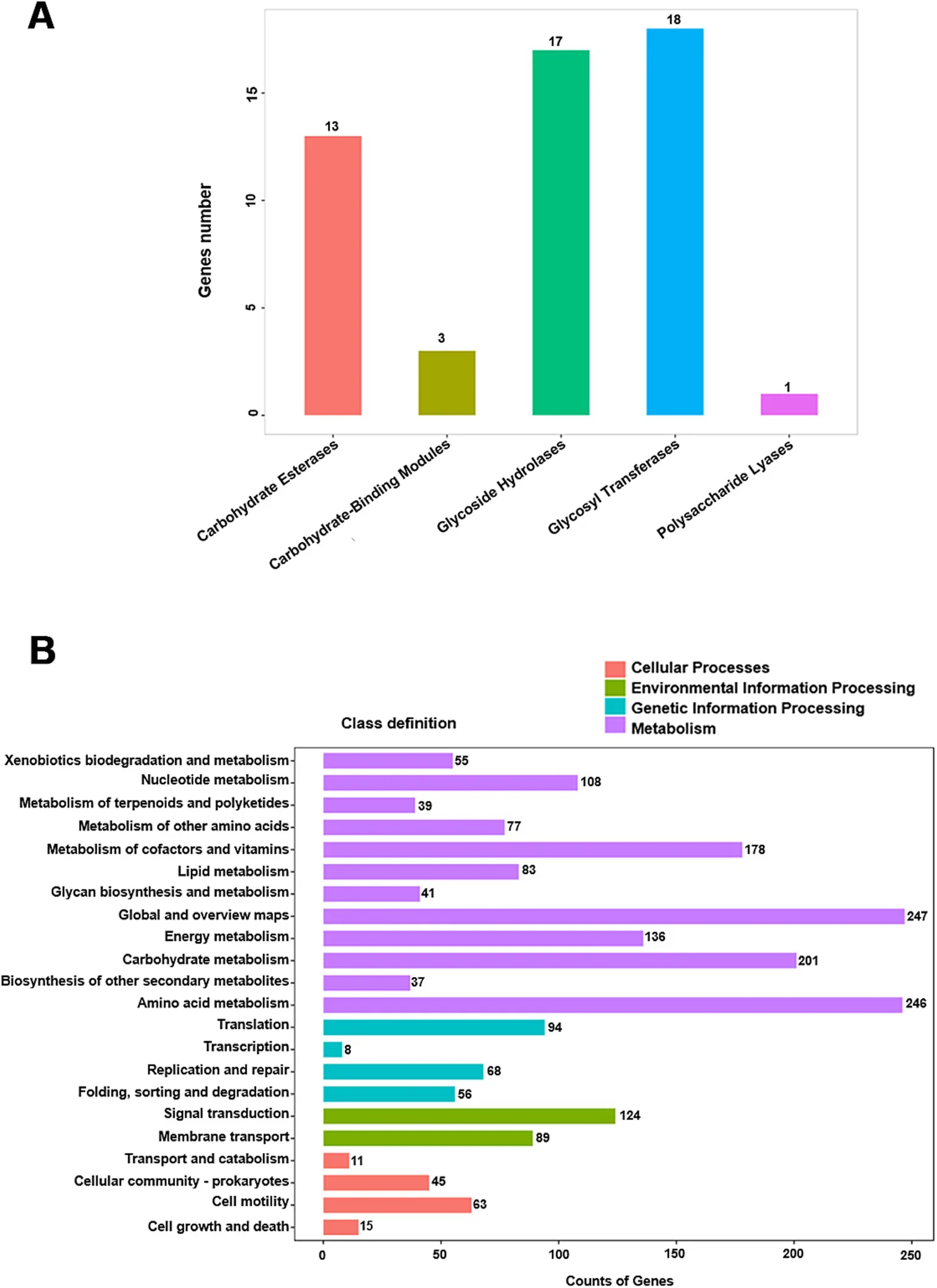
Distribution of CAZy and KEGG annotation classes. **(A)** CAZy annotation classification distribution map. The CAZy classification is represented on the abscissa, whereas the number of genes annotated to each respective classification is depicted on the ordinate. **(B)** KEGG annotation statistics chart at Level 2. The horizontal axis denotes the number of genes, and the vertical axis represents the name of the Level 2 pathway. The numbers adjacent to the right side of each column indicate the quantity of genes annotated to that Level 2 pathway.

Notably, 66% of the genome remains unassigned to RAST subsystems. Analysis of carbohydrate-active enzymes revealed 18 glycosyl transferase (GT) genes and 17 glycosyl hydrolase (GH) genes (Figure 3A and Supplementary Table S3). KofamKOALA analysis revealed the presence of major bacterial metabolic versatility in strain WY3 (Figure 3B and Supplementary Tables S4, S5). Genes involved in amino acid and carbohydrate metabolism were particularly prominent, suggesting that strain WY3 may possess efficient nutrient uptake systems. Furthermore, genes associated with the biodegradation of chlorinated aromatic hydrocarbons, including *atoB*, *fadI*, *fadJ*, *adhP*, *hmgL*, and *gabD*, were identified, suggesting potential roles in environmental remediation.

### 3.3 Genetic relatedness and pan-genome analysis

**Figure 4:**
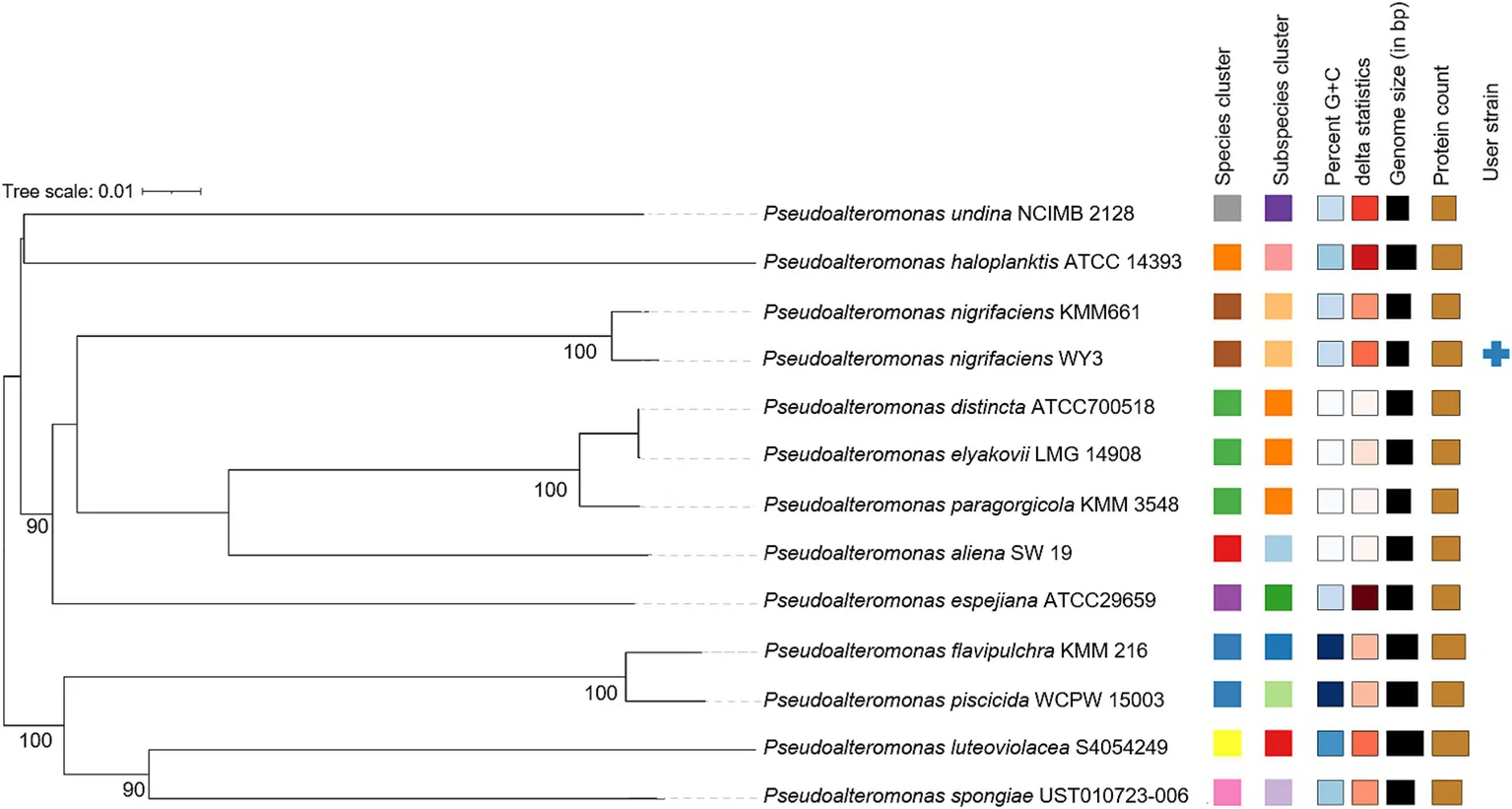
The phylogenetic tree was constructed based on the whole genome via the Genome–BLAST distance phylogenetic method (GBDP) tool. The branch lengths were scaled according to the GBDP distance Formula d5.

The elucidation of phylogenetic relationships among strains, as determined through the Genome Blast Distance Phylogeny (GBDP) method, placed strain WY3 in a cluster alongside *P. nigrifaciens* KMM661^T^ (Figure 4). The ANI values of the genome of *P. nigrifaciens* FME68^T^ exceeded 98% (Table 2), whereas the DDH values between strain WY3 and other strains within *P. nigrifaciens* surpassed 85% (Table 3), indicating a very close relationship between WY3 and *P. nigrifaciens*.

**Table 2:** Calculated ANIb values between available genomes of WY3 and type strains.

Species	WY3	FME 68^T^	KMM 661^T^	NBRC 103036^T^	NCTC 10691^T^	ATCC 14393^T^
WY3	100					
FME 68^T^	98.39	100				
KMM 661^T^	98.38	99.99	100			
NBRC 103036^T^	98.37	100	99.99	100		
NCTC10691^T^	96.29	99.99	95.79	95.89	100	
ATCC 14393^T^	75.74	75.76	75.91	75.82	75.85	100

**Table 3:** The dDDH values are provided along with their confidence intervals (C.I.) for the GBDP formula 2.

Query	Subject	d4	C.I. d4
WY3	*Pseudoalteromonas nigrifaciens* NBRC 103036	87.8	[85.3–89.9]
WY3	*Pseudoalteromonas nigrifaciens* FME68	87.6	[85.1–89.8]
WY3	*Pseudoalteromonas nigrifaciens* NCTC10691	87.5	[84.9–89.7]
WY3	*Pseudoalteromonas nigrifaciens* KMM661	87.5	[84.9–89.7]
WY3	*Pseudoalteromonas haloplanktis* ATCC 14393	20.8	[18.6–23.3]

**Figure 5:**
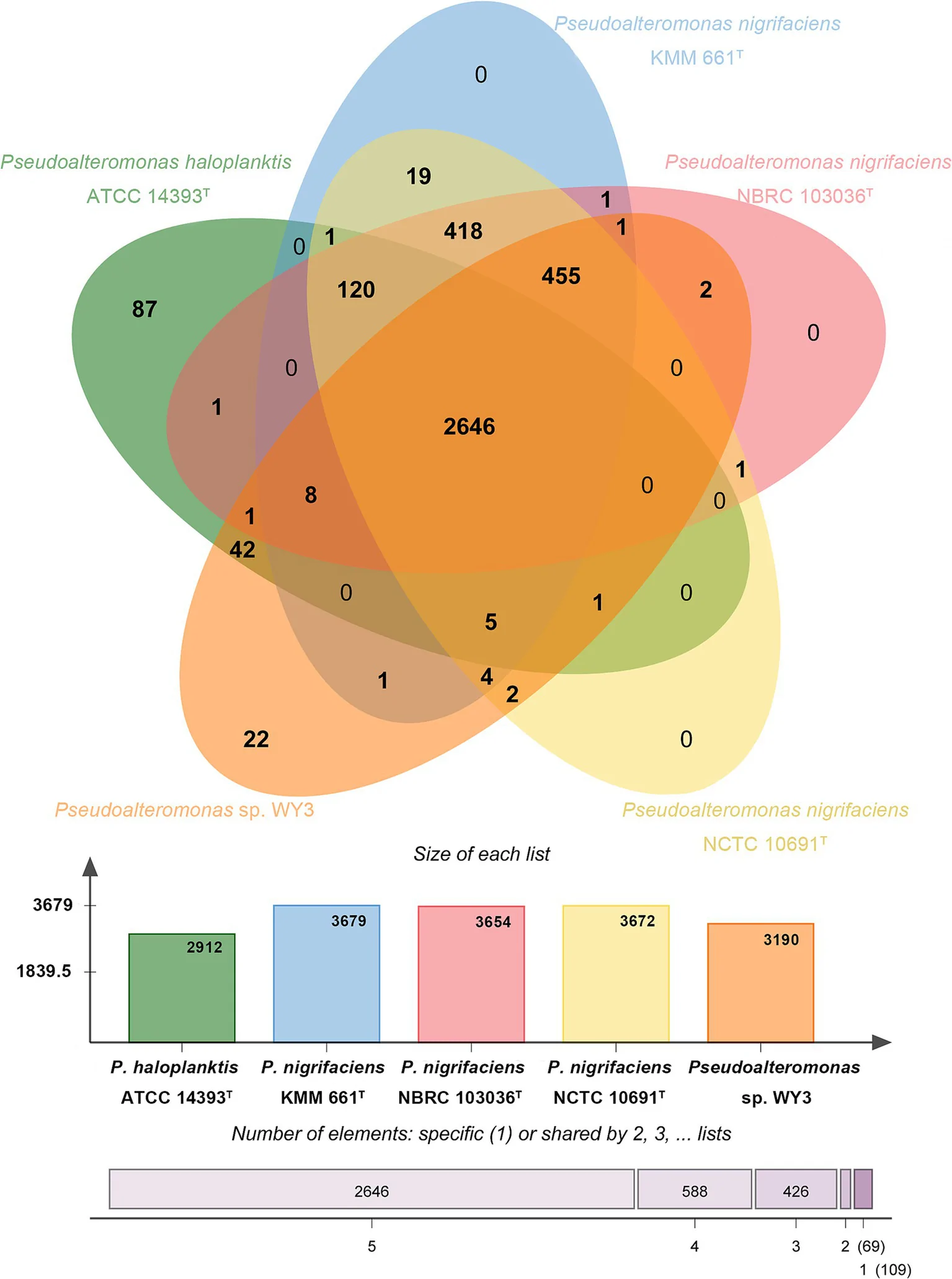
The Venn diagram and the bar graph depict the comparative genomics analysis among the genomes of *Pseudoalteromonas* sp. WY3, *P. nigrifaciens* NCTC 10691^T^, *P. nigrifaciens* NBRC 103036^T^, *P. nigrifaciens* KMM 661^T^ and *P. haloplanktis* ATCC 14393^T^, showing both shared and unique orthologous genes clusters.

Using comparative pangenome analysis, we provided a comprehensive overview of the genomic content of the strain *Pseudoalteromonas* sp. WY3 and related strains within the genus *Pseudoalteromonas*, as depicted in a Venn diagram (Figure 5). The analysis revealed 2,624 shared gene clusters among five *Pseudoalteromonas* strains, with strain WY3 possessing 28 unique gene clusters. The core genome, comprising 1,361 orthologous clusters (34.54%), was enriched in genes related to essential bacterial processes. The unique gene clusters in strain WY3 were predominantly associated with carbohydrate metabolism and the response to stimuli, suggesting specialized adaptations to its Antarctic krill host and cold environment.

### 3.4 Secondary metabolites

**Figure 6:**
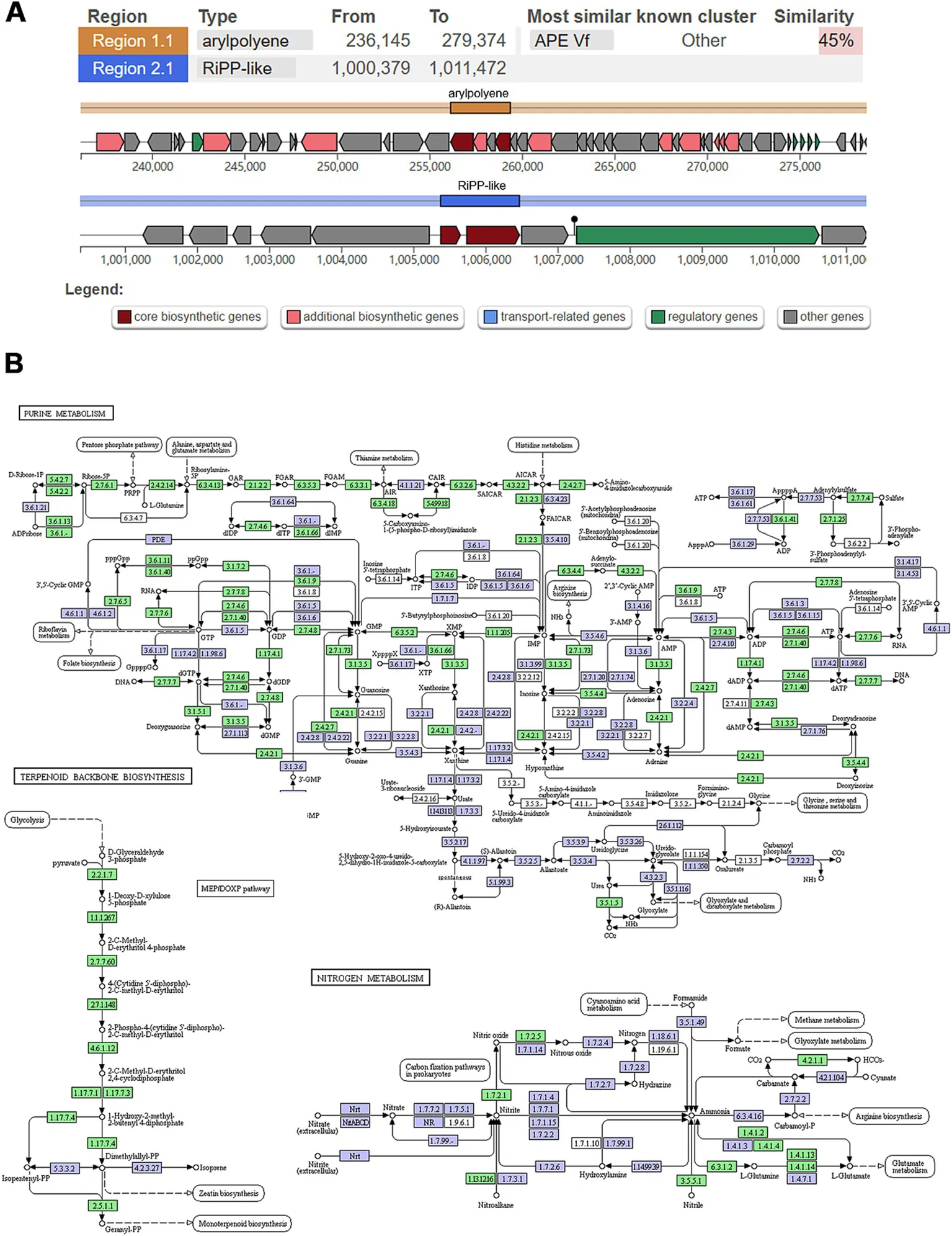
Analysis of secondary metabolism and genomic islands in WY3 genome. **(A)** Biosynthesis-related gene clusters were predicted via antiSMASH. **(B)** KEGG pathways related to purine metabolism, terpenoid backbone biosynthesis, and nitrogen metabolism. Points represent nodes in the pathway diagram, which are mainly composed of genes, metabolites, and upstream and downstream adjacent pathways. They correspond to three different shapes of symbols (rectangle, dot, and obtuse rectangle). The metabolic map, represented by the blue boxes, is a comprehensive and detailed illustration based on existing knowledge. It serves as a general reference. The green boxes indicate genes or enzymes that are unique to this species.

AntiSMASH analysis identified two biosynthesis-related gene clusters (BGCs) in strain WY3. Cluster 1 (236,145–279,374 nucleotides), related to aryl polyene (APE) biosynthesis, displayed 45% similarity to the known BGC 0000837 (Figure 6A). Strain WY3 contained the *ApeH* gene, encoding an acylcarbamoyl peptidase involved in APE biosynthesis. Cluster 2, spanning nucleotides 1,000,379 to 1,012,146, was identified as belonging to RiPP-like related clusters. These orphan biosynthesis-related gene clusters (BGCs) showed no discernible homology to known counterparts, suggesting the potential production of novel ribosomally synthesized and post-translationally modified peptides (RiPPs). The genome also harbored the *ubiC* gene, encoding chorismate lyase, which is involved in the biosynthesis of 4-hydroxybenzoic acid (4-HBA), a compound with known antimicrobial activity and potential anticancer properties.

A detailed KEGG analysis was conducted on strain WY3 to explore its secondary metabolic capabilities. The analysis revealed a diverse array of genes involved in various metabolic pathways, highlighting the bacterium’s robust metabolic capacity and potential (Supplementary Table S6). The results show the presence of genes encoding enzymes crucial for amino acid biosynthesis, such as aspartate aminotransferase (*aspC*), aspartate kinase (*lysC*), and glutamate 5-kinase (*proB*), indicating that the strain can synthesize essential amino acids like aspartate, lysine, and proline. Moreover, the presence of genes related to the synthesis of aromatic amino acids, such as chorismate mutase/prephenate dehydrogenase (*tyrA*) and histidinol-phosphate aminotransferase (*hisC*), suggests that the WY3 strain is capable of synthesizing a broad spectrum of amino acids. The analysis also identified genes involved in the biosynthesis and modification of secondary metabolites, including tryptophan halogenase (*prnA*) and lysine decarboxylase (*ldcC*). Additionally, we found a gene encoding penicillin amidase (E3.5.1.11), which is involved in the hydrolysis of penicillin and its derivatives. The presence of these genes implies that strain WY3 may be involved in producing and modifying bioactive compounds, potentially contributing to its ecological interactions and adaptability in the Antarctic environment. Furthermore, the analysis revealed genes involved in carbohydrate metabolism, such as glucose-1-phosphate thymidylyltransferase (*rfbA*) and dTDP-glucose 4,6-dehydratase (*rfbB*), which are involved in cell wall biosynthesis; phosphoglucomutase (pgm), which is crucial for sugar metabolism; and glucokinase (*glk*), a key enzyme in glucose utilization. These enzymes indicate a versatile carbohydrate metabolism that may be essential for the bacterium’s survival in the cold Antarctic environment. Efficient carbohydrate metabolism is crucial in cold conditions as it allows for rapid energy production and the synthesis of cryoprotectants, helping the bacterium maintain cellular function at low temperatures.

The KEGG pathway analysis also highlights interconnected pathways such as purine metabolism, terpenoid backbone biosynthesis, and nitrogen metabolism (Figure 6B). The prominently featured purine metabolism pathway underscores WY3’s ability to synthesize, degrade, and salvage purine nucleotides, ensuring efficient energy management and genetic stability under nutrient-limited conditions. The MEP/DOXP pathway, essential for isoprenoid biosynthesis, suggests a robust mechanism for maintaining membrane fluidity and integrity, which is crucial for cold adaptation. Additionally, the nitrogen metabolism pathway indicates the bacterium’s ability to efficiently assimilate and utilize nitrogen, further demonstrating its metabolic flexibility and ecological success in extreme habitats.

Overall, these findings highlight *Pseudoalteromonas* WY3’s potent metabolic capabilities, particularly in synthesizing and managing key biomolecules essential for survival in harsh conditions. The well-developed pathways for purine metabolism, terpenoid biosynthesis, and nitrogen metabolism not only underscore the strain’s adaptation to its extreme environment but also its significant biosynthetic potential. This positions *Pseudoalteromonas* WY3 as a promising candidate for biotechnological applications, including the production of cold-adapted enzymes and valuable bioactive compounds.

### 3.5 Islands of genome

**Figure 7:**
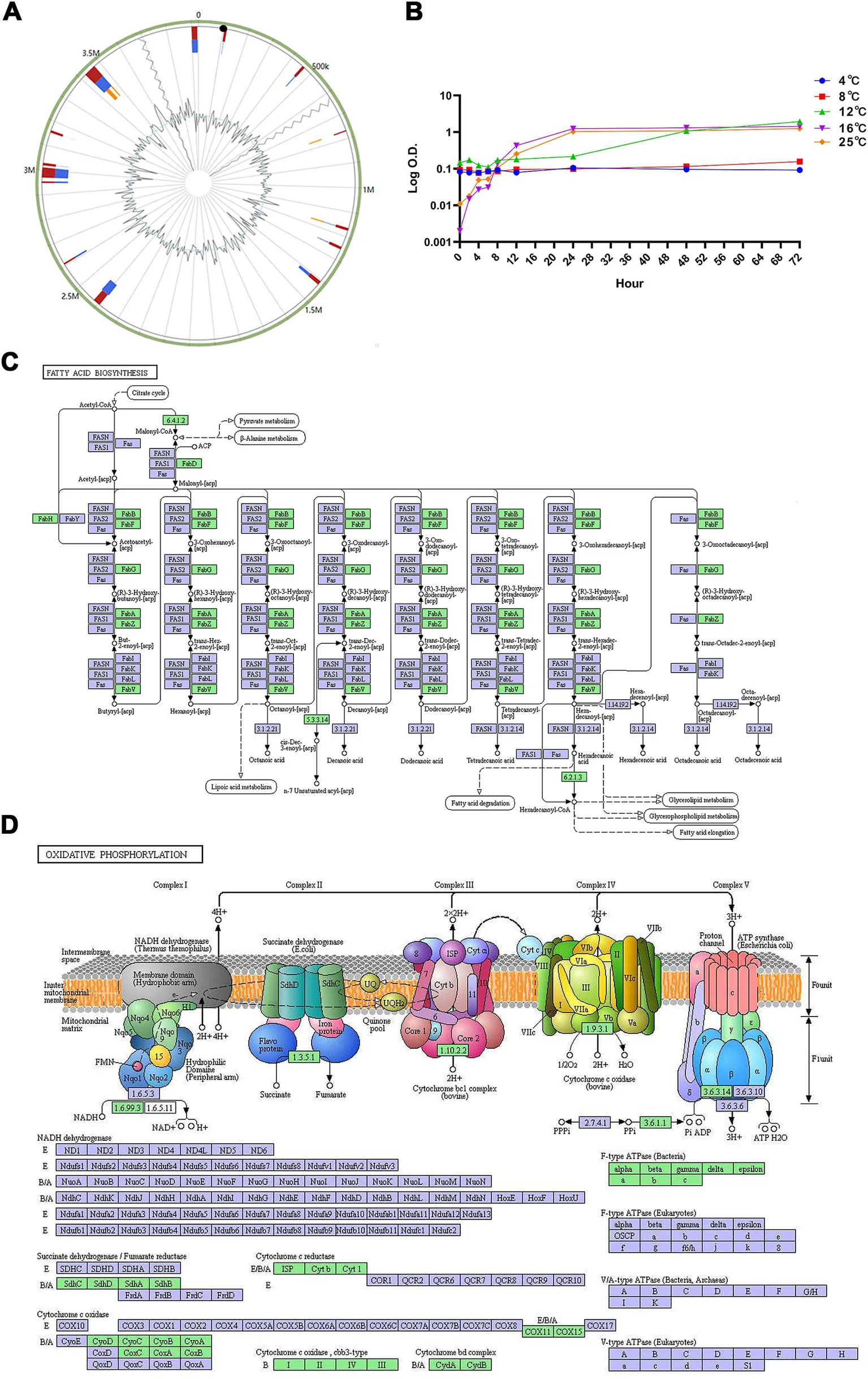
**(A)** The Genomic Islands (GIs) in strain WY3 were predicted. The red color represents the prediction made via an integrated approach, whereas the blue color depicts the results obtained via IslandPath-DIMOB. Orange represents the genomic islands predicted via SIGI-HMM. **(B)** Growth curves of *Pseudoalteromonas* sp. strain WY3 at various temperatures. The growth of strain WY3 was monitored at 4°C (blue circles), 8°C (red squares), 12°C (green triangles), 16°C (purple inverted triangles), and 25°C (orange diamonds) over a 72-h period. Growth was assessed by measuring the optical density at 600 nm (OD600) at regular intervals. The logarithm of OD600 is plotted against incubation time. Each data point represents the mean of triplicate measurements. **(C)** Fatty acid metabolism and **(D)** oxidative phosphorylation metabolic pathways mapped using KEGG analysis. Points represent nodes in the pathway diagram, which are mainly composed of genes, metabolites, and upstream and downstream adjacent pathways. They correspond to three different shapes of symbols (rectangle, dot, and obtuse rectangle). The metabolic map, represented by the blue boxes, is a comprehensive and detailed illustration based on existing knowledge. It serves as a general reference. The green boxes indicate genes or enzymes that are unique to this species.

Seventeen genomic islands, comprising 306 genes, were predicted in the genome of strain WY3 (Figure 7A). These islands included 131 genes encoding hypothetical proteins and 12 encoding mobile element proteins. No resistance or virulence factor genes were identified, suggesting the low pathogenic potential of strain WY3.

### 3.6 Environmental adaptability

To assess the cold adaptation of strain WY3, growth experiments were conducted over 72 h at temperatures ranging from 4°C to 25°C, revealing its psychrotrophic nature (Figure 7B). Our analysis using the Ratkowsky model demonstrated that the bacteria exhibit remarkable cold tolerance, with a predicted minimum growth temperature (*T*_min_) of −1.2°C (Table 4), indicating potential growth near freezing conditions. While growth was minimal at 4°C and 8°C (with growth rates of 0.0011/h and 0.0021/h, respectively), we observed significant growth at higher temperatures. The predicted optimal growth temperature (*T*_opt_) of approximately 24.8°C aligns well with the observed data, where the fastest growth occurred between 16°C and 25°C (with growth rates of 0.1428/h and 0.1478/h, respectively). This indicates that while these bacteria can grow at low temperatures, they thrive most efficiently at or slightly below room temperature. We estimated the theoretical maximum growth temperature (*T*_max_) at 36.5°C. This wide growth temperature range (−1.2°C to 36.5°C) highlights the strain’s exceptional cold adaptability and metabolic flexibility, which may play a crucial role in its symbiotic relationship with Antarctic krill.

**Table 4:** Predicted minimum, maximum, and optimum growth temperatures for WY3.

Preincubation temp (°C)	Observed growth rate *r* (h^−1^)	Predicted temp range (°C)		
		*T*_min_	*T*_opt_	*T*_max_
4	0.0011	−1.2	24.8	36.5
8	0.0021			
12	0.0449			
16	0.1428			
25	0.1478			

To further elucidate the molecular basis of strain WY3’s cold adaptation, we performed a comprehensive genomic analysis, which revealed a diverse array of genes and pathways that contribute to its remarkable adaptability to low-temperature environments. Strain WY3 displays a range of adaptations that enable it to thrive in cold environments (Table 5). It harbors 10 genes encoding cold shock proteins (*CspA*), which are crucial for stabilizing RNA and proteins during sudden temperature drops. Additionally, the strain includes molecular chaperones such as *IbpA*, *HSPE1*, *HSPD1*, and components of the *GroEL*/*GroES* system, which help maintain protein integrity under cold stress. The strain’s antioxidant defense system is also robust, featuring superoxide dismutase (*SOD2*), three catalase genes (*katE*, *CAT*, *catB*), and three glutathione peroxidase (*GPx*) genes, which collectively neutralize reactive oxygen species commonly encountered in cold conditions. The presence of three glyceraldehyde-3-phosphate dehydrogenase (*GAPDH*) genes further enhances the bacterium’s stress response capabilities. While lacking the common alginate system, WY3 possesses genes for betaine aldehyde dehydrogenase (*gene3474*) and choline dehydrogenase (*gene3475 and gene3476*), enabling it to convert choline to betaine for osmoprotection. Notably, the genome contains 15 peptidyl-prolyl cis-trans isomerase (PPIase) genes, indicating a robust capacity for protein folding under cold stress, similar to that observed in the cold-adapted *P. haloplanktis* TAC 125^T^ strain (Table 6). These features highlight WY3’s potential as a model for cold adaptation studies and as a candidate for recombinant protein production in cold environments.

**Table 5:** List of key genes related to cold adaptation discovered in the genome of strain WY3.

Gene_id	Identity	KO	Gene_name	Description
gene1179	69/69 (100.00)	K03704	*cspA*	cold shock protein (beta-ribbon, CspA family)
gene1180	69/69 (100.00)	K03704	*cspA*	cold shock protein (beta-ribbon, CspA family)
gene1181	69/69 (100.00)	K03704	*cspA*	cold shock protein (beta-ribbon, CspA family)
gene2426	72/72 (100.00)	K03704	*cspA*	cold shock protein (beta-ribbon, CspA family)
gene2987	67/67 (100.00)	K03704	*cspA*	cold shock protein (beta-ribbon, CspA family)
gene3214	69/69 (100.00)	K03704	*cspA*	cold shock protein (beta-ribbon, CspA family)
gene3509	68/68 (100.00)	K03704	*cspA*	cold shock protein (beta-ribbon, CspA family)
gene3510	67/69 (97.10)	K03704	*cspA*	cold shock protein (beta-ribbon, CspA family)
gene3511	69/69 (100.00)	K03704	*cspA*	cold shock protein (beta-ribbon, CspA family)
gene3512	69/69 (100.00)	K03704	*cspA*	cold shock protein (beta-ribbon, CspA family)
gene3188	148/148 (100.00)	K04080	*ibpA*	molecular chaperone IbpA
gene3189	148/148 (100.00)	K04080	*ibpA*	molecular chaperone IbpA
gene3190	148/148 (100.00)	K04080	*ibpA*	molecular chaperone IbpA
gene0863	95/95 (100.00)	K04078	*groES, HSPE1*	chaperonin GroES
gene0864	535/535 (100.00)	K04077	*groEL, HSPD1*	chaperonin GroEL
gene2956	145/145 (100.00)	K04564	*SOD2*	superoxide dismutase, Fe-Mn family
gene2392	69/70 (98.57)	K03781	*katE, CAT, catB, srpA*	catalase
gene2393	396/410 (96.59)	K03781	*katE, CAT, catB, srpA*	catalase
gene3727	508/511 (99.41)	K03781	*katE, CAT, catB, srpA*	catalase
gene1084	163/163 (100.00)	K00432	*gpx*	glutathione peroxidase
gene1555	181/182 (99.45)	K00432	*gpx*	glutathione peroxidase
gene2913	162/164 (98.78)	K00432	*gpx*	glutathione peroxidase
gene2200	480/480 (100.00)	K00134	*GAPDH, gapA*	glyceraldehyde 3-phosphate dehydrogenase
gene2784	333/334 (99.70)	K00134	*GAPDH, gapA*	glyceraldehyde 3-phosphate dehydrogenase
gene3750	336/337 (99.70)	K00134	*GAPDH, gapA*	glyceraldehyde 3-phosphate dehydrogenase

**Table 6:** Prolyl isomerases encoded in the genomes of representative extremophilic and mesophilic methanogenic archaea and their environmental temperature (Feller, 2018).

Strain	Temperature	PPiase genes
*Colwellia psychrerythraea* 34H	<0°C	18
*Pseudolateromonas haloplanktis* TAC 125	<0°C	15
*Pseudomonas extremaustralis* 14–3	<0°C	14
*Escherichia coli* K12	37°C	10
*Thermus thermophilus* HB8/HB27	65°C	4
*Thermotoga maritima* MSB8	85–90°C	1
*Aquifex aeolicus* VF5	85–90°C	1

Further validation of WY3’s cold adaptation was achieved through an in-depth KEGG analysis of its genome. This analysis identified two critical pathways essential for cold adaptation: fatty acid metabolism (ko00061) and oxidative phosphorylation (ko00190), the latter of which playing a significant role in the bacterium’s antioxidant defense (Figures 7B,C). The fatty acid metabolism pathway includes enzymes such as *FabD*, *FabH*, and various *FabB* and *FabF*, which are vital for synthesizing and modifying fatty acids. These enzymes are crucial for maintaining membrane fluidity, particularly at low temperatures. The presence of *FabA* and *FabZ* suggests the bacterium synthesizes unsaturated fatty acids to enhance cold tolerance. Moreover, the oxidative phosphorylation pathway analysis (Figure 7D) revealed a complete set of electron transport chain complexes (I-V), including NADH dehydrogenase, succinate dehydrogenase, cytochrome bc1 complex, cytochrome c oxidase, and ATP synthase. The presence of a fully functional electron transport chain indicates a robust energy production system, which is essential for maintaining cellular function during cold stress. Collectively, these findings demonstrate that WY3 is highly adapted to cold environments, with its ability to modify membrane composition through fatty acid metabolism enhancing cold tolerance and its complete oxidative phosphorylation system ensuring efficient energy production crucial for survival in extreme cold. Strain WY3’s genomic features and growth characteristics highlight its remarkable cold adaptation, making it a promising model for studying cold adaptation mechanisms and a potential candidate for recombinant protein production in low-temperature environments.

## 4 Discussion

Oceans, which cover more than 70% of the Earth’s surface, contain a range of extreme environments, particularly in polar regions that are characterized by low temperatures, high pressure, and high salinity (Brett et al., 2020). These conditions create habitats for a diverse range of microorganisms, particularly in polar regions, which play pivotal roles in global biogeochemical cycles (Clark et al., 2023). Antarctica, renowned for its frigid environments, has yielded numerous microorganisms that exhibit robust cold adaptation mechanisms (Vincent, 2000). The Antarctic krill, a keystone species in the Southern Ocean ecosystem, serves as a host for a rich microbiome that significantly contributes to marine bacterial diversity (Croxall et al., 1999; Troussellier et al., 2017). Despite their ecological importance, host-associated microbial communities in polar environments are often understudied, underscoring the importance of studies such as this one, which aim to broaden our understanding of microbial diversity and interactions in extreme environments.

Our study focused on *Pseudoalteromonas* sp. WY3, a strain isolated from Antarctic krill. The genus *Pseudoalteromonas*, which is part of the marine gamma-proteobacteria, is known for its environmental adaptability and ability to thrive in a variety of extreme habitats (Hwang et al., 2016). This makes *Pseudoalteromonas* an excellent model system for investigating microbial environmental adaptations to polar environments. Genomic analysis revealed that strain WY3 belongs to the species *P. nigrifaciens* based on ANI and dDDH values that surpass species delimitation thresholds (Chun et al., 2018). The close genetic relationship between WY3 and other *P. nigrifaciens* strains suggests that WY3 has adapted well to its polar environment, likely through processes such as horizontal gene transfer. The genomic islands identified in WY3 provide additional insights into its adaptability. These islands suggest that the strain may have acquired adaptive traits through horizontal gene transfer, further enhancing its ability to thrive in polar environments. This evolutionary process could be crucial for understanding how microorganisms in extreme environments develop resilience to environmental stressors.

The genomic analysis of WY3 revealed the presence of unique gene clusters related to carbohydrate metabolism and the stress response. Representing specialized adaptations that enable survival in the cold, nutrient-limited conditions of the Southern Ocean. The identification of two BGCs in WY3, including one related to APE biosynthesis, suggests the potential for novel metabolite production. APEs are known to increase bacterial resistance to external threats and improve survival, which could be crucial in extreme environments (Johnston et al., 2021). Additionally, the presence of these BGCs implies that WY3 could contribute to microbiota formation and microbial protection in marine invertebrates, potentially assisting in host defense against pathogens (Offret et al., 2016; Bowman, 2007).

The growth characteristics of WY3, as elucidated by our experiments and the Ratkowsky model analysis, provide fascinating insights into the adaptability of this *Pseudoalteromonas* species. The remarkable temperature range over which WY3 can grow suggests a highly specialized adaptation to the Antarctic marine environment and, more specifically, to the microenvironment provided by its krill host. The estimated minimum growth temperature (*T*_min_) of −1.2°C indicates that WY3 has evolved mechanisms to maintain cellular functions near freezing conditions, a trait crucial for survival in the extreme Antarctic waters. This extreme cold tolerance is likely a key factor in WY3’s ability to persist as a symbiont of Antarctic krill, which inhabit waters that can reach temperatures close to freezing (Flores et al., 2012). While growth at 4°C and 8°C was minimal, the ability to proliferate at these temperatures demonstrates WY3’s capacity to maintain metabolic activity under the cold conditions frequently encountered by krill. This suggests that WY3 can remain active and potentially provide benefits to its host even during the coldest periods of the Antarctic year.

The optimal growth temperature (*T*_opt_) of approximately 24.8°C, coupled with rapid growth observed at 16°C and 25°C, illustrates WY3’s metabolic flexibility. This broad temperature adaptability may be particularly advantageous for Antarctic krill, which can experience significant temperature variations due to vertical migration in the water column and seasonal changes (Tarling and Thorpe, 2014). WY3’s ability to thrive across this temperature range could allow it to maintain its symbiotic relationship with krill throughout these environmental fluctuations. The extended lag phase observed at lower temperatures, particularly evident in the growth curve at 12°C, likely reflects a period of physiological adjustment. During this time, WY3 may be optimizing its cellular machinery for low-temperature conditions through mechanisms such as altering membrane lipid composition, producing cold-shock proteins, and modifying RNA and protein synthesis processes (Chattopadhyay, 2006). These adaptations could potentially benefit the krill host by providing cold-active enzymes or other molecules that aid in low-temperature physiological processes.

The discovery of secondary metabolite gene clusters in strain WY3 underscores its potential to produce novel bioactive compounds, with significant implications for biotechnology. The low similarity of the BGCs of WY3 to known clusters suggests the potential to uncover new metabolites with unique properties, which could be valuable in medicine, agriculture, and environmental management. One such example is the strain’s ability to produce 4-HBA, a compound linked to antimicrobial and anticancer activities. The genome analysis revealed the presence of the *ubiC* gene, which encodes chorismate lyase, an enzyme that catalyzes the conversion of chorismate into pyruvate and 4-HBA, marking the initial step in the ubiquinone biosynthetic pathway in gram-negative bacteria. Previous studies have isolated 4-HBA from *Pseudoalteromonas* species, where it exhibited notable antimicrobial properties (Yu et al., 2012). Additionally, 4-HBA produced by *P. haloplanktis* TAC 125^T^, a strain isolated from Antarctica, has shown potential to induce cancer cell responses (Sannino et al., 2018), further emphasizing the biotechnological importance of strain WY3 in the production of compounds with therapeutic potential.

Another significant aspect of the WY3 genome is the high abundance of peptidyl-prolyl cis isomerases (PPIs), enzymes crucial for maintaining protein folding under cold stress. These PPIs are like those found in the cold-adapted *P. haloplanktis* TAC 125^T^, indicating a robust capacity for cold adaptation (Parrilli et al., 2021). Interestingly, the elevated levels of PPIs in *P. haloplanktis* TAC 125^T^ are associated with high recombinant protein production, which suggests that WY3 may also be a promising candidate for recombinant protein production in biotechnological applications (Colarusso et al., 2022). Additionally, the presence of cold shock proteins and antioxidant enzymes, including catalase, superoxide dismutase, and glutathione peroxidase, further underscores the ability of the strain to survive and function in extremely cold environments by mitigating oxidative stress.

The KEGG analysis of the *Pseudoalteromonas* WY3 strain provides valuable insights into its powerful metabolic capacity and potential for the production of novel bioactive compounds. The presence of genes involved in the biosynthesis of a wide range of amino acids and secondary metabolites suggests that this bacterium has the genetic potential to produce a diverse array of metabolites with biotechnological applications. The identification of genes related to tryptophan halogenase and lysine decarboxylase is particularly noteworthy, as these enzymes are known to participate in the synthesis of bioactive compounds with antimicrobial, anticancer, and other pharmacological properties (Caboche et al., 2010). These findings highlight the potential of the WY3 strain as a source of novel secondary metabolites that could be exploited for drug discovery and other biotechnological applications. Moreover, the presence of genes involved in carbohydrate metabolism indicates that the WY3 strain has a versatile metabolic capacity, which may contribute to its ability to adapt to the challenging conditions of the Antarctic environment. The synthesis and modification of various sugar derivatives are essential for bacterial cell wall biosynthesis and the production of extracellular polysaccharides (Whitfield and Trent, 2014), which may play a role in the bacterium’s survival and interactions with its host, the Antarctic krill. To fully exploit the metabolic potential of the *Pseudoalteromonas* WY3 strain, further experimental work is needed to characterize the bioactive compounds produced by this bacterium and to elucidate their biosynthetic pathways.

Despite these promising findings, this study has several limitations that require further investigation. The biotechnological potential of *Pseudoalteromonas* sp. WY3, particularly with respect to the novel metabolites predicted by genomic analysis, remains largely unexplored. Experimental verification of these metabolites, especially those related to APE biosynthesis, is crucial for understanding their biosynthetic pathways and evaluating their applications in bioremediation, natural product discovery, and industrial biotechnology. Additionally, the interactions between WY3 and its krill host are not fully understood, leaving a gap in our knowledge of host–microbe coevolution in extreme environments. Future research should explore the potential probiotic effects of WY3 on Antarctic krill and other marine organisms, investigate its suitability for recombinant protein production, particularly for proteins requiring low-temperature expression, and assess its antioxidant properties for possible biotechnological or pharmaceutical applications. Comprehensive studies on the role of WY3 in host health and metabolism, as well as the broader microbial ecology of polar regions, will further enhance our understanding of these unique microbial communities and their potential applications.

## 5 Conclusion

In this study, the whole genome of *Pseudoalteromonas* sp. WY3 was assembled, and its genomic characteristics were thoroughly analyzed via bioinformatic approaches. Through comparative genomics, intricate genomic characteristics of this strain were elucidated, revealing its close phylogenetic relationship with *P. nigrifaciens* and prognosticating its potential biological functionalities. These findings are expected to enrich the microbial resource database and offer novel insights into the symbiotic interplay between culturable microorganisms and their hosts in extreme environments. Furthermore, they offer a theoretical foundation for further research and development involving *Pseudoalteromonas.*

## Data Availability

The datasets presented in this study can be found in online repositories. The names of the repository/repositories and accession number(s) can be found in the article/Supplementary material.
