# Correction: Genomic insights into the cold adaptation and secondary metabolite potential of *Pseudoalteromonas* sp. WY3 from Antarctic krill

**DOI:** 10.3389/fmicb.2026.1942243

**Published:** 2026-09-21

**Authors:** Yuanyuan Wang, Jinxuan Xie, Zhengqi Feng, Linbo Ma, Wenbo Wu, Changjun Guo, Jianguo He

**Affiliations:** 1 State Key Laboratory for Biocontrol/Southern Marine Science and Engineering Guangdong Laboratory (Zhuhai), Guangdong Province Key Laboratory of Aquatic Economic Animals & Guangdong Provincial Observation and Research Station for Marine Ranching of the Lingdingyang Bay, School of Marine Sciences, Sun Yat-sen University, Guangzhou, China; 2 Key Laboratory of the East China Sea and Oceanic Fishery Resources Exploitation, Ministry of Agriculture, East China Sea Fisheries Research Institute, Shanghai, China

**Keywords:** Antarctic krill, marine bacterium, *Pseudoalteromonas*, genomic analysis, extremophiles

There were mistakes in Table 1 as published. The G + C content and several genome annotation statistics, including the numbers of total genes and protein-coding genes, were incorrectly reported. The corrected Table 1 appears below.

**Table 1:** Genome statistics of the *Pseudoalteromonas* sp. WY3.

Attribute	Value	% of total
Genome size (bp)	3,926,696	100
DNA coding region (bp)	3,315,162	84.43
DNA G + C (bp)	1,570,447	39.99
DNA scaffolds	3	—
Total genes	4,234	100
Protein-coding genes	4,107	97.00
RNA genes	127	3.00
Genes assigned to COGs	3,059	74.48
Genes assigned Pfam domains	14	0.34
Genes with signal peptides	368	8.96
Genes with transmembrane helices	943	22.96

The G + C content, total number of genes, and number of coding sequences were incorrectly reported. A correction has been made to section **3.2 Genomic characteristics**, Paragraph 1:

“The genome assembly of strain WY3 included 3 scaffolds, with a total length of 3,926,696 base pairs and a G + C content of 39.99% (Table 1). A total of 4,234 genes were identified, including 4,107 coding sequences (Figure 2A).”

The number of genes encoding cold shock proteins (*CspA*) was incorrectly reported. A correction has been made to the section **3.6 Environmental adaptability**, Paragraph 2:

“It harbors 10 genes encoding cold shock proteins (*CspA*), which are crucial for stabilizing RNA and proteins during sudden temperature drops.”

The original version of this article has been updated.

